# Study on Spatial-Temporal Change of Urban Green Space in Yangtze River Economic Belt and Its Driving Mechanism

**DOI:** 10.3390/ijerph182312498

**Published:** 2021-11-27

**Authors:** Chunyu Chen, Linglan Bi, Kuanfan Zhu

**Affiliations:** 1 School of Architecture and Design, Southwest Jiaotong University, Chengdu 611756, China; chenchunyu@my.swjtu.edu.cn; 2 Jiangxi Provincial Research Institute of Territorial Space Survey and Planning, Nanchang 330009, China; zhukf07@lzu.edu.cn

**Keywords:** urban green space, spatial–temporal change, drive mechanism, geodetector, YREB

## Abstract

Urban green space plays an important role in beautifying the environment, improving the quality of life of residents, and promoting sustainable urban development. Rapid urbanization has led to great changes in the spatial structure and layout of urban green space. It is urgent to put forward the sustainable development strategy of green space through the research on the change of urban green space. Based on the geographical spatial differences of urban green space and integrating the factors of economy, society, industry, land use, and the environment, we constructed a research framework of “space-supply-demand” integration of urban green space by GI and geodetector methods, and we conducted an empirical study on the spatial–temporal changes of urban green space and its driving mechanism in prefecture-level cities along the Yangtze River Economic Belt in China. First, the urban green space along the Yangtze River Economic Belt is concentrated in spatial distribution, while uneven development appears in urban greening among the zones. Second, the influence of different factors on urban green space change varies greatly and can be divided into three types: key factors, important factors, and auxiliary factors. The driving mechanism of the spatial distribution of urban green space supply and demand is quite different, but urban population and commercial service facilities land are their key influence factors, having a comprehensive influence on the spatial–temporal changes of urban green space. Third, the factors are classified into three categories of high, medium, and low levels according to the mean of interacting forces; in particular, the factors of per capita GDP, utility land, industrial smoke (dust) emissions, and other factors have a very strong interactive effect with other factors. Fourth, according to the spatial distribution characteristics of urban green space and its driving mechanism, this paper puts forward planning and policy suggestions, providing reference for other areas to deal with the green space change.

## 1. Introduction

The urban green space (UGS) is an important part of urban ecological space, which can provide important ecosystem services (ESs). For example, it can better air quality [1] and lower the surface temperature [2,3,4] to improve the urban thermal environment and cope with climate change [5,6], while reducing runoff [7] and controlling flood [8], to improve the sustainability of ecological environment; meanwhile, it can help protect biodiversity [9] and improve the sustainability of the ecosystem [10]; in addition, it can promote the health of residents [11] and increase social cohesion [12,13], to improve the quality of life of residents and increase the sustainability of living environment [14]. The rapid development of urbanization has altered the structure and pattern of urban green space, affecting the quantity and quality of urban green space, which may lead to decreasing the supply capacity of ecosystem services, having a negative influence on urban environmental quality and human well-being. Therefore, it is urgent to put forward the sustainable development strategy of green space through the research on the change of urban green space, which can help enhance the ability of cities to cope with environmental changes, and it is of great significance for maintaining the stability of urban green space systems and realizing the sustainable development of urban ecological environment.

There has been a consensus of scholars at home and abroad to carry out the quantitative and qualitative and multidisciplinary research on urban green space, and it is also a hot spot in the fields of ecology, geography, landscape ecology, environmental science, forestry, and landscape architecture [15]. The research on urban green space carried out by scholars currently focuses on how urban green space improves the urban thermal environment [16,17,18], the efficiency of urban green space use [19,20], association between urban greenery and travel propensity [21,22], and the connection with urban morphology [23,24]. The frontier of urban green space morphology includes studies on urban green space planning, studies on urban green space patterns and urban landscape patterns, and studies on strategies, planning management, and solutions for the sustainable development of urban green space [25]. The sustainable development strategy of urban green space based on the study of urban green space change and its driving factors has gradually become a hot topic. Most of the urban green space change studies feature the analysis of the characteristics and patterns of the green space change in a particular city or region [26,27,28,29], next are the studies emphasizing the importance of green space change in urban planning and decision making [30,31,32]. Policy influence is most mentioned in studies on driving factors of green space change [33,34,35]; it is followed by urban population and land use [36,37,38], real estate development and increased demand for recreational space [39], industrial development, economic growth, demand desire, and public investment [40], and other factors. The methods used by scholars for the above studies mainly include statistical analysis [41], spatial analysis [42], remote sensing monitoring [43], and simulation prediction based on the Cellular Automata Model [44]; in contrast, the research on urban green space change [45] and driving factors [46] using geographic detectors is still in its infancy.

The research in this field is usually based on a certain city, and there is a lack of variety in the research scale; as a result, there are overly microscopic results that do not accurately reflect the spatial–temporal change patterns at a regional scale. Therefore, this paper focuses on the cities along the Yangtze River Economic Belt at the prefecture level and above, hoping to discover more universal patterns. There is no quantitative study on the effect of multiple influencing factors and the interaction effect between factors; therefore, the interaction study between multiple influencing factors of urban green space change based on the geodetector method in this paper will accurately reflect the result of the synergistic effect of multiple factors on urban green space change. Then, we develop a research framework of integration to the “space-supply-demand” integration of urban green space, which can provide more scientific and reasonable decision support for urban green space system planning, subsequently providing a reliable basis for urban planning and governmental decision making.

This paper focuses on the study of the following issues: (1) How to analyze the spatial and temporal evolution of urban green space to discover its current characteristics and evolutionary patterns with the continuous progress of urbanization? (2) How to quantitatively measure the influence of each factor and evaluate the interaction effects between the factors as the urban green space change is influenced by a larger number of factors? (3) What reasonable planning and design strategies and policy suggestions should be put forward according to the above research? Therefore, this paper analyzes the spatial and temporal evolution of urban green space in a prefecture-level city in the Yangtze River Economic Belt of China to quantify the magnitude of the influencing factors and the strength of the interaction effects between them, in an attempt to explore the driving mechanisms of urban green space change and further propose planning and policy recommendations, with a view to providing a reference for decision making on sustainable urban ecological development in China and other similar countries.

## 2. Materials and Methods

### 2.1. Study Area: The Yangtze River Economic Belt (YREB)

**Figure 1:**
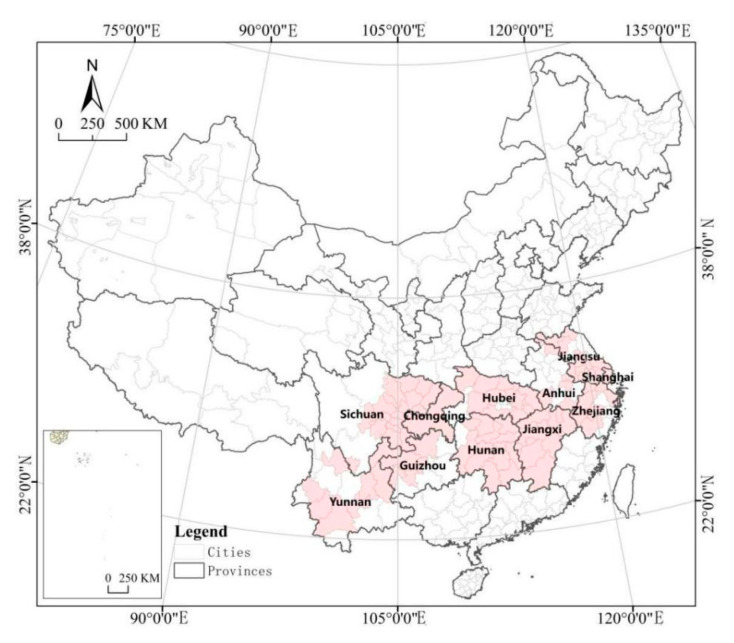
Study area.

The study is based on the cities along the Yangtze River Economic Belt in China (see Figure 1), covering 11 provinces and cities, that is, Shanghai, Jiangsu, Zhejiang, Anhui, Jiangxi, Hubei, Hunan, Chongqing, Sichuan, Yunnan, and Guizhou, accounting for 21.4% of China’s total area and more than 40% of China’s population and GDP. The Yangtze River Economic Belt stretches across the eastern, central, and western regions of China, with unique advantages and great development potential. Since the reform and opening up, the Yangtze River Economic Belt has developed into one of the regions with the strongest comprehensive strength and the greatest strategic support in China. The “Belt and Road” initiative, the Beijing–Tianjin–Hebei Coordinated development initiative, and the Yangtze Economic Belt initiative are the three national development strategies. In September 2014, The State Council made a plan to build the Yangtze River Economic Belt into an inland river economic belt with global influence, a coordinated development belt of interaction and cooperation between the East and the West, a comprehensive opening-up belt along the coastal areas, rivers, and borders, and a pioneering demonstration belt of ecological civilization construction. The National Development and Reform Commission and the Forestry Administration made arrangements in February 2016 to further strengthen afforestation and promote the construction of a green ecological corridor along the Yangtze River Economic Belt. In November 2018, the State Council explicitly called for the coordinated development of the upper, middle, and lower reaches of the Yangtze River and high-quality development of the regions along the golden waterway, with ecological priority and green development as the guide.

With the rapid urbanization process in China, the urban green space area and park area also show a steady growth. The green space change in cities along the Yangtze River Economic Belt in the new period is related to the development of urban ecological civilization and the improvement of people’s quality of life, so it is of great theoretical significance and practical value to study its spatial–temporal change and driving factors.

### 2.2. Research Methods

#### 2.2.1. Gini Index: GI

The Gini index is used to evaluate the fairness of social resources distribution, such as measuring the fairness of public transportation service, medical resources, and mineral resources distribution [47]. The Gini index of green space distribution can accurately reflect the uniformity and fairness of green space distribution in a specific region [48,49]. Its value ranges from 0 to 1. A larger value indicates a more concentrated distribution of green space, while a smaller value indicates a more even distribution, with 0.4 considered as its warning line. According to the research method of Xue [50], in this paper, a Gini index greater than 0.4 means there is a large gap, indicating a concentrated distribution of green space; a value greater than 0.6 means there is a great large gap, indicating a rather concentrated distribution of green space (see Table 1).

#### 2.2.2. Kernel Density Analysis: ARCGIS

The calculation method for nuclear density analysis is based on the first law of geography [51], and it is used to calculate the density of elements in their surrounding areas. It is a means to transform the set of point elements (vector data) into raster data. The function of the kernel density is to fit each point or line into a smooth cone surface by calculating the value per unit area of the point or line element based on the kernel function.

This paper visualizes the geographical distribution of dependent variable indicators of the urban green space based on the kernel density analysis method of ARCGIS. In order to reflect the spatial distribution of urban green space scientifically and macroscopically, the kernel density of the study area is classified into five grades: extremely high, high, medium, low, and quite low according to the natural breaks [52].

#### 2.2.3. Geodetector

The geodetector model is a statistical method to effectively detect the spatial heterogeneity of geographic phenomena and reveal their driving factors, which are used to detect the degrees of influence of multiple factors under different spatial units and their inter-relationships. This model has been now widely used in land use, regional economy, ecological environment, and other fields to explore the driving forces of various social phenomena and their interactions [53,54,55]. In this study, two modules, factor detection and interaction detection, were used to study the driving factors of green space change and their interactions. Factor detection helps to identify the influence factors, while interaction detection helps to explain the interaction of the influencing factors on the dependent variable.

By comparing whether there is significant spatial consistency between a factor and changes in geographic matters, the factor detector determines whether the factor has a determinant role in the occurrence and development of the geographic matters while further detecting the extent to which the factor explains the spatial heterogeneity of the geographic matters. The model is used for analysis of the influence q of driving factors ($X_{i}$) on urban green space ($Y_{i}$) change; the range of influence q value is [0, 1]. At some significance test level (typically 0.05), a larger q value indicates that there is a stronger influence of the selected indicators ($X_{i}$) on urban green space ($Y_{i}$) change; otherwise, there is a weaker influence.

The interaction detector is the biggest advantage of the geodetector over other statistical methods, which can empirically demonstrate the interaction influence of multiple factors. Through analysis, it can work out whether the joint action of influencing factors will increase or decrease the dependent variable *Y*; that is, the factors have an interactive or independent influence on the dependent variable. The interaction between influencing factors $X_{i}$ and $X_{j}$ will produce five relationships (see Table 2).

### 2.3. Index Selection

According to the availability of data and comparability of spatio-temporal analysis, two indicators were used as dependent variables ($Y_{i}$) in this paper. The built-up urban green area ($Y_{1}$) can be used to measure urban development level and green space supply, and park area ($Y_{2}$) can be used to measure residents’ quality of life and green space demand.

Urban green space is the result of many factors, including nature, economy, society, policy, industrial structure, infrastructure and public service facilities, and ecological environment. Different factors will produce more complex relations or influence changes in the process of superposition. Although the change of urban green space area is the result of the comprehensive action of natural geography, social economy, and policy system factors [56], physical geography is the basic condition of urban green space change, policy system is the means of regulating and controlling urban green space change, and social economy is a major driving factor in the short-term urban green space change [57]. The green space coverage of the built-up area is more likely to reflect the influence of urbanization than the influence of climate or geographical factors [58]. Therefore, this paper focuses on socio-economic drivers. Based on the research experience of Cheng [15] et al., as well as the data accessibility and integrity, we chose 15 indicators from five areas of economic development, social conditions, industrial structure, land use structure driving force, and ecological environment as independent variables to explore the driving mechanism of urban green space change (see Table 3).

We assume in the construction of the indicator system that GDP and per capita GDP represent the influence of urban economic development on urban green space supply [59], the investment completed in the current year reflects the potential and strength of government support for urban green space development [60], the local general public budget expenditure reflects the government’s efforts to improve people’s livelihood, the population size and the urban area represent the potential scale of urban green space demand [61], the average wage of on-job employees reflects the consumption capacity of and the demand for urban green space [62]; the output value of secondary industry and industrial land represent the real economy, the output value of tertiary industry and commercial service land reflect the supporting capacity of services, the lands for road traffic facilities and public facilities reflect the service capacity and quality of public facilities and infrastructure, and the density of drainage pipes in built-up areas and the industrial smoke (dust) emissions [63] reflect the quality of the ecological environment. The geographical detection and analysis of independent and dependent variables can reveal the connections of the spatial–temporal characteristics of urban green space change with urban economy, society, industry, land use, and environment, and they can provide a basis for relevant policy design.

The rapid urbanization process in China constitutes the basic background and conditions for the spatio-temporal pattern of urban green space and its evolution. Economic development is an important driving force for the expansion of urban green space area, so GDP, per capita GDP, the urban population, and urban area are essential independent variables. To a certain extent, industrial structure promotes the development of urban landscaping and the improvement of urban living environment, and it accordingly drives the development and improvement of the tertiary industry, indicating that the ecological environment and industrial economic structure complement each other. Therefore, the output value of the secondary industry, output value of the tertiary industry, industrial land, and land for commercial service facilities are selected as independent variables. The increasing government investment provides a basic guarantee for the improvement of urban green space construction, so the investment completed in the current year and local general public budget expenditure are selected as independent variables. With the improvement of living standards, people have higher requirements for infrastructure and urban environment, so the average wage of on-job employees, land for road transportation facilities, utility land, density of drainage pipes in built-up areas, and industrial tobacco (powder) dust emissions are selected as independent variables.

### 2.4. Research Steps

**Figure 2:**
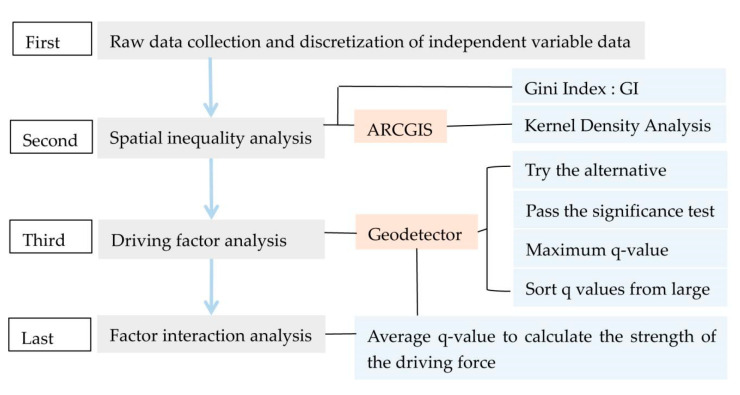
The technical flowchart of this study.

This study mainly includes four steps (see Figure 2). The first step is raw data collection and discretization of independent variable data. Prepare a complete table of raw data based on the data published by the relevant statistical websites, discrete the continuous data of the independent variables using Python, and to eliminate artificial influence, classify the data of the independent variables into 10 categories (2–11) by the quantile method. The second step is spatial inequality analysis. Calculate the Gini index of the dependent variable in order to find the uniformity of urban green space distribution; then, analyze the spatial–temporal change of the dependent variable by ARCGIS to discover the spatial and temporal distribution of urban green space. The third step is driving factor analysis. Import the source data of the dependent variable and the discrete data of the independent variable (the result of step 1) into geodetector and calculate the analysis results. Select the better alternatives in the results of 10 categories, select the one with the largest q-value that meets the same or higher significance level as the final solution based on the significance test as a criterion for the credibility of the results, and determine the strength of the explanatory power of the independent variables by ranking them according to the size of the q-value to obtain the influence of driving factors. The fourth step is factor interaction analysis. Find the mean of q-values of the independent variables that pass the significance test, calculate the strength of the driving forces, and analyze the interaction effects of the driving factors to further reveal the driving mechanisms and policy implications of urban green space change.

### 2.5. Data Sources

The statistical data were collected from the China Urban Construction Statistical Yearbook (2015–2019), China City Statistical Yearbook (2016–2020), and some missing data were collected from the statistical yearbook of the concerned cities (2016–2020). There are two reasons for choosing the period from 2015 to 2019: (1) it helps to maintain the consistency of the statistical caliber of the data according to the feasibility of the data; (2) since 2015, the YREB has developed at a higher level, and China has entered a new period of ecological civilization construction with urban development into the stock era from incremental. There are 110 prefecture-level cities along the Yangtze River Economic Belt, and only 93 were analyzed for driving factors due to the limited availability of data.

## 3. Results

### 3.1. Spatial Inequality Analysis

#### 3.1.1. Spatial Difference Analysis: GI

In the stock era, urban green space also presents a concentration with the gradual stabilization of urbanization development. The Gini indexes of $Y_{1\;}$ and $\;Y_{2\;}$ are 0.53, 0.52, 0.52, 0.53, 0.53, and 0.53, 0.59, 0.59, 0.58, 0.49, respectively, all greater than 0.4 and less than 0.6, indicating that the distribution of urban green space is concentrated and nonuniform. The Gini index of $Y_{1\;}$ from 2015 to 2019 was stable with no obvious changes, while the Gini index of $\;Y_{2\;}$ showed a clear change of first increasing and then decreasing, indicating that the park area experienced the change in concentration from increasing first and then decreasing with the development of the city. With the improvement of the quality of life, there is an increase in the uniformity of park area, and it will be gradually uniform, that is, relatively fair to urban residents.

#### 3.1.2. Spatial–Temporal Change Analysis: GIS

**Figure 3:**
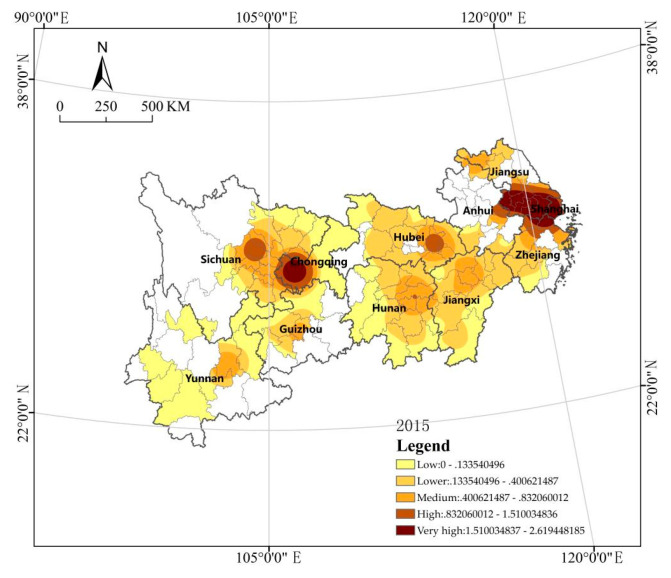

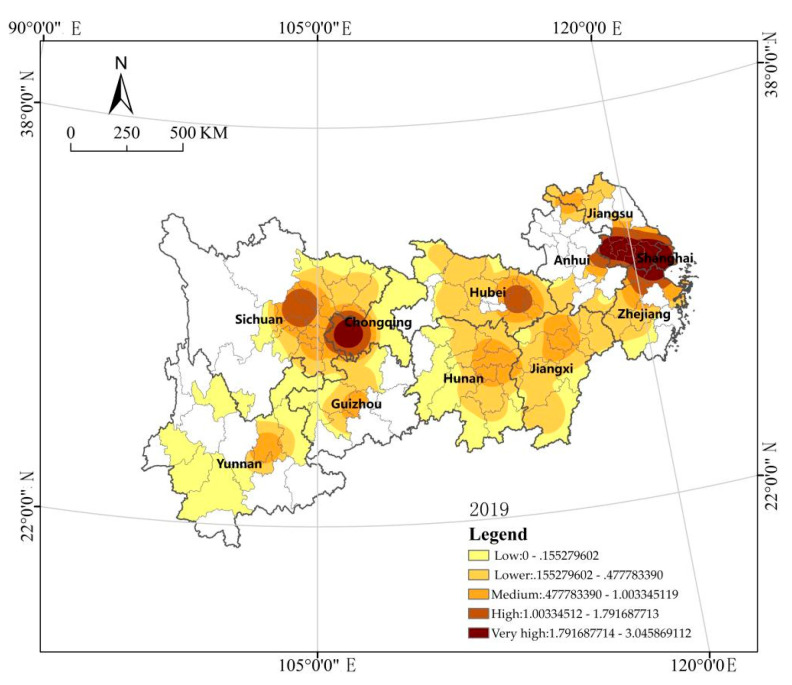
Kernel density analysis of the urban green area (2015–2019).

Kernel density analysis can reflect the spatial agglomeration of analysis targets. The urban green area ($Y_{1}$) is unevenly distributed in space (see Figure 3). According to the figure, the urban green area in 2015 and 2019 presents the characteristics of spatial distribution with the Yangtze River Delta and Chengdu–Chongqing urban agglomeration as the core, provincial capital cities as nodes, and a significantly larger area in the central–eastern region than in the western region. It reflects the higher level of urban green space construction usually in economically developed cities, such as the coastal areas in the Yangtze River Delta and the Chengdu–Chongqing urban agglomeration region. In contrast, southwest China has a low level of urban green space construction due to its unfavorable geographical location for economic development, such as the mountainous areas of Yunnan, Guizhou, and Sichuan [64]. The spatial–temporal change is characterized by a significantly larger green area in Chengdu and a significantly smaller one in Changsha according to the comparison between 2015 and 2019. The reason is that since Chengdu proposed to build a park city in 2018, there has been a significant increase in the urban green area. In contrast, the central and southern region, where Changsha is located, has a high level of greening construction, and its green area has reached a certain scale. Due to the limited land area in the region, the increase in green space area is greatly restricted, and therefore, they place more focus on improving the quality of green space in green space development [64].

**Figure 4:**
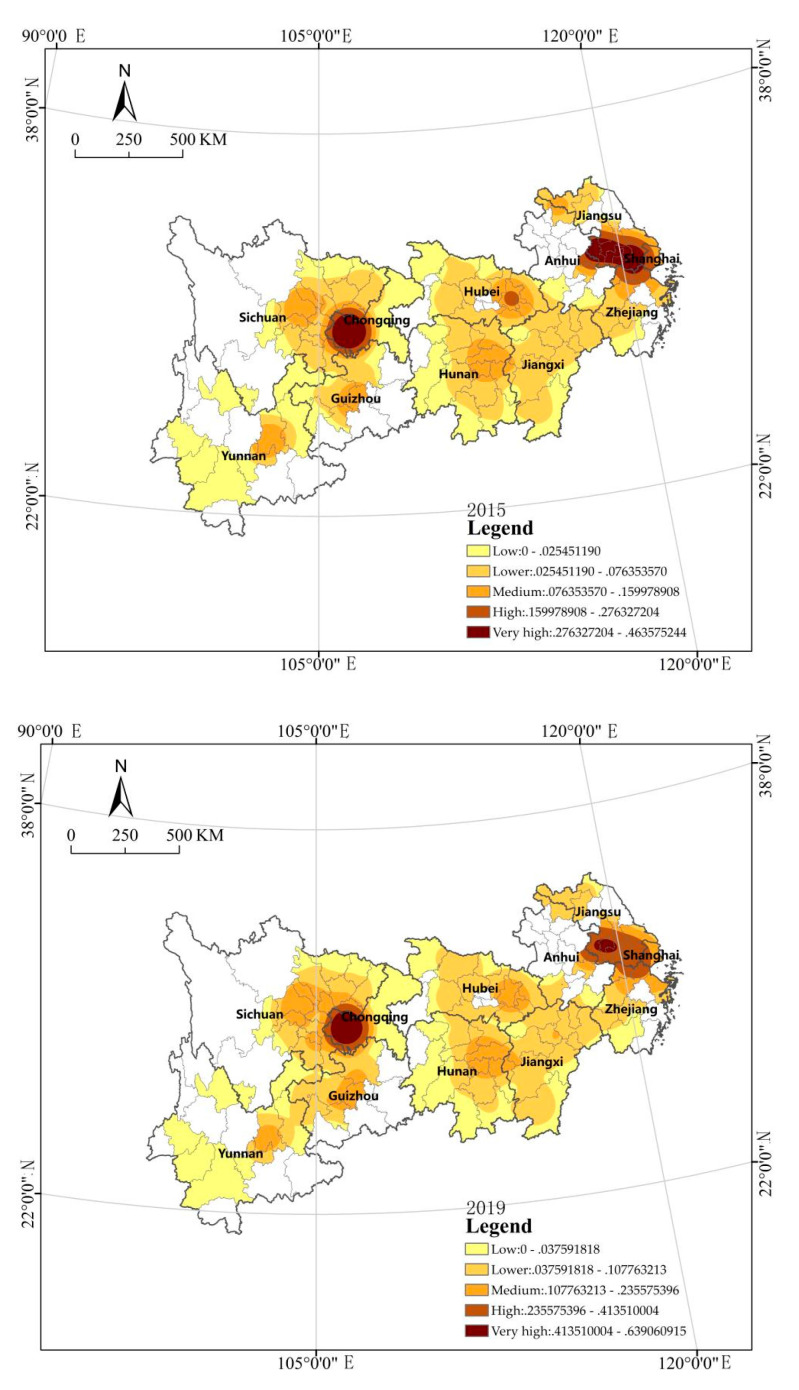
Kernel density analysis of the park area (2015–2019).

The urban park area ($\;Y_{2}$) is unevenly distributed in space (see Figure 4). According to the figure, the urban park area in 2015 presents the characteristics of spatial distribution with Chongqing and the Yangtze River Delta as the core, provincial capital cities as nodes, and a significantly larger area in the central–eastern region than in the western region. The distinctive feature is the large park area in four cities, Nanjing, Shanghai, Chongqing, and Wuhan, which have all held the China International Garden and Flower Expo (CIGF EXPO). CIGF EXPO is the highest level and largest international event in China’s landscaping industry, and such large-scale exhibitions have an obvious role in promoting the increase in park area. The urban park area in 2019 presents the characteristics of spatial distribution with Chongqing and Nanjing as the core, provincial capital cities as nodes, and a significantly larger area in the central–eastern region than in the western region. The spatial–temporal change is characterized by a clear trend of decrease in Wuhan and the Yangtze River Delta, especially Shanghai, according to the comparison between 2015 and 2019, which is probably due to greater focus on improving the quality of green space when it reaches a certain scale, or more land use for squares, leading to a decrease in statistics after changes in classification standards.

### 3.2. Driving Factor Analysis

#### 3.2.1. Urban Green Area ($Y_{1}$)

**Figure 5:**
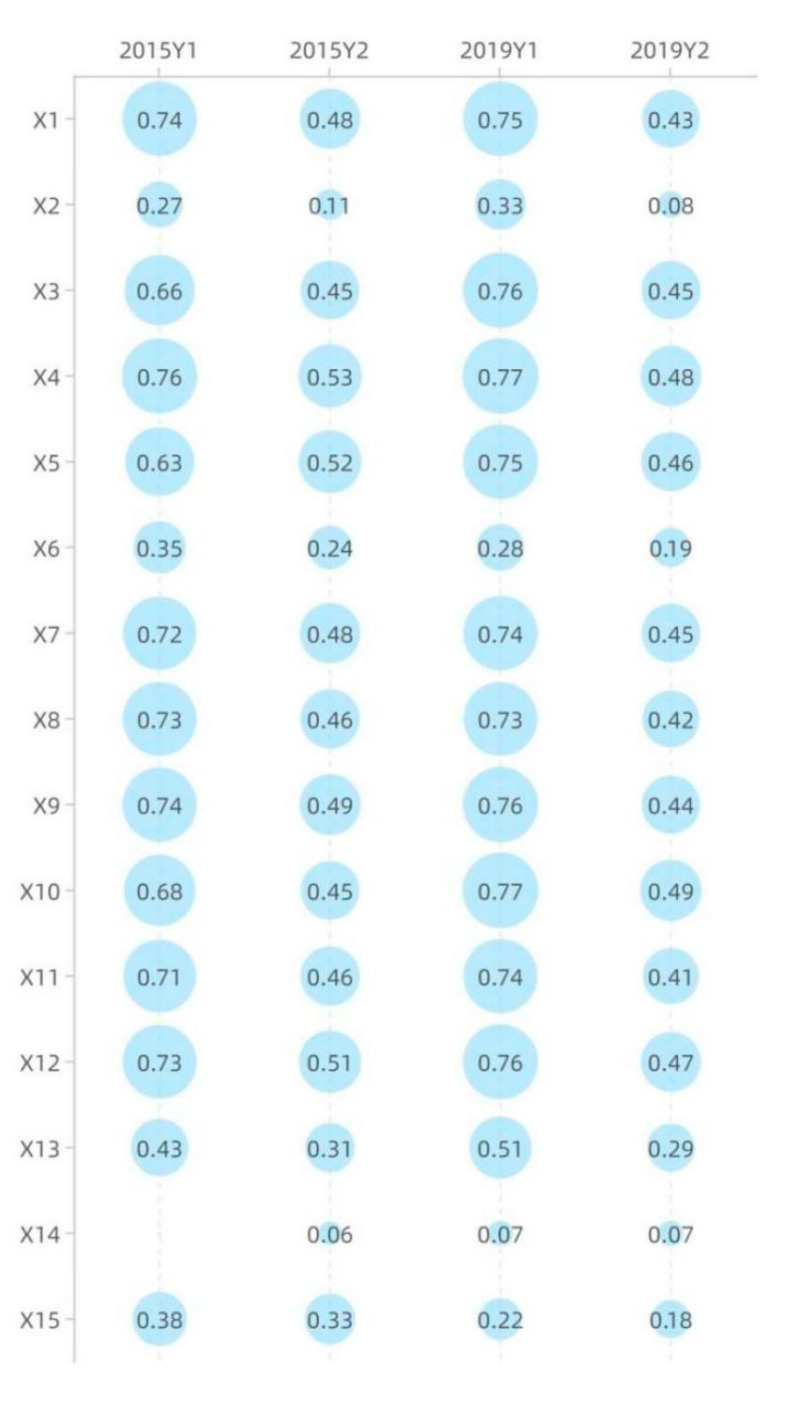
The q-value of the factor detector result.

**Figure 6:**
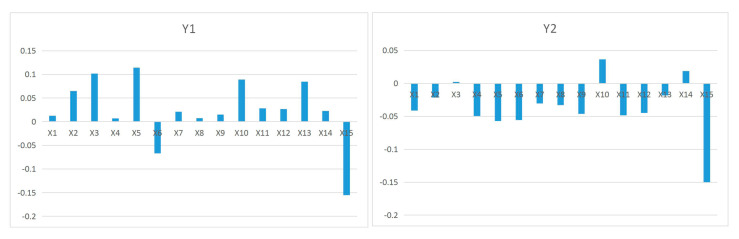
Change of factor influence from 2015 to 2019.

**Figure 7:**
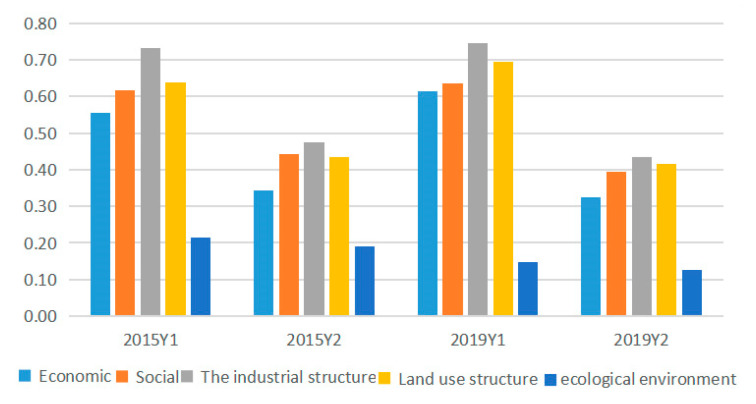
Analysis of driving forces in five aspects.

At some significance test level (typically 0.05), a larger q-value indicates that there is a stronger influence of the selected indicators ($X_{i}$) on urban green area ($Y_{1}$) change. In 2019, all the driving factors ($X_{i}$) passed the significance test of 5% and a more stringent level. At the significance level of 5%, the driving factors were ranked in the order of intensity depending on the q-value as $X_{10}$ > $X_{4}$ > $X_{3}$ > $X_{12}$ > $X_{9}$ > $X_{1}$ > $X_{5}$ > $X_{7}$ > $X_{11}$ > $X_{8}$ > $X_{13}$ > $X_{2}$ > $X_{6}$ > $X_{15}$ > $X_{14}$. In 2015, $X_{14}$ failed to pass the significance test, while the remaining factors could pass the significance test of 5% and a more stringent level. At the significance level of 5%, the driving factors were ranked in the order of intensity depending on the q-value as $X_{4}$ > $X_{1}$ > $X_{9}$ > $X_{12}$ > $X_{8}$ > $X_{7}$ > $X_{11}$ > $X_{10}$ > $X_{3}$ > $X_{5}$ > $X_{13}$ > $X_{15}$ > $X_{6}$ > $X_{2}$ (see Figure 5). There were 13 factors that increased in force from 2015 to 2019, including $X_{5}$, $X_{3}$, $X_{10}$, $X_{13}$, and $X_{2}$ with a larger rise; and two decreased, with $X_{15}$ weakening more than $X_{6}$ (see Figure 6). It should be noted that $X_{14}$ evolved to pass the significance test of 5%. The driving forces of urban green area ($Y_{1}$) in 2015 and 2019 were in the same order, specifically, industrial structure > land use structure > social > economic > the ecological environment (see Figure 7).

#### 3.2.2. Park Area ($Y_{2}$)

At some significance test level (typically 0.05), a larger q-value indicates that there is a stronger influence of the selected indicators ($X_{i}$) on park area ($Y_{2}$) change. In 2019, all the driving factors ($X_{i}$) passed the significance test of 5% and a more stringent level. At the significance level of 5%, the driving factors were ranked in the order of intensity depending on the q-value as $X_{10}$ > $X_{4}$ > $X_{12}$ > $X_{5}$ > $X_{3}$ > $X_{7}$ > $X_{9}$ > $X_{1}$ > $X_{8}$ > $X_{11}$ > $X_{13}$ > $X_{6}$ > $X_{15}$ > $X_{2}$ > $X_{14}$. In 2015, $X_{14}$ could only pass the loose significance test of 10%, while the rest of the factors could pass the significance test of 5% and a more stringent level. At the significance level of 5%, the driving factors were ranked in the order of intensity depending on the q-value as $X_{4}$ > $X_{5}$ > $X_{12}$ > $X_{9}$ > $X_{7}$ > $X_{1}$ > $X_{11}$ > $X_{8}$ > $X_{10}$ > $X_{3}$ > $X_{15}$ > $X_{13}$ > $X_{6}$ > $X_{2}$ (see Figure 5). There were three factors that increased in force from 2015 to 2019, in the ranking order of $X_{10}$ > $X_{14}$ > $X_{3}$, with $X_{15}$ weakening more than the other two. It should be noted that $X_{14}$ evolved to pass the significance test of 5% (see Figure 6). There is a very large difference in the ranking of driving forces in the park area ($Y_{2}$) between 2015 and 2019, with the former being the industrial structure > social > land use structure > economic > the ecological environment, and the latter being the industrial structure > land use structure > social > economic > the ecological environment (see Figure 7).

### 3.3. Factor Interaction Analysis

**Figure 8:**
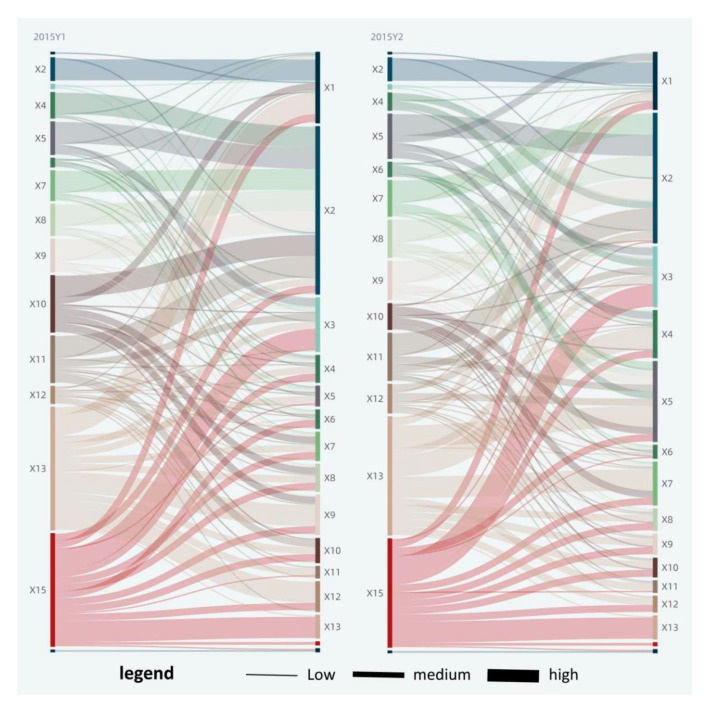
Analysis of interaction detector in 2015.

**Figure 9:**
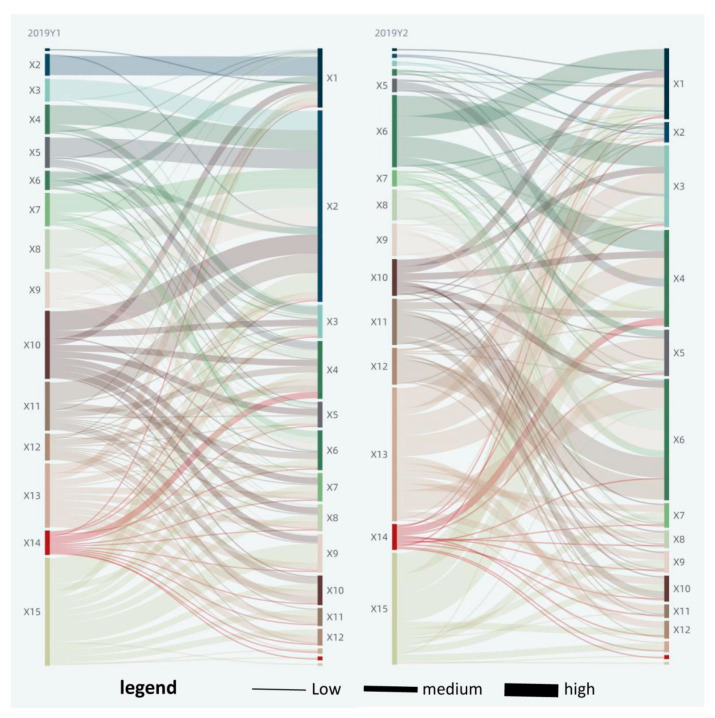
Analysis of interaction detector in 2019.

The value of the interaction force of factors ranges from 0 to 1, and an increasing value indicates that the interaction force is getting stronger. At the significance level of 5%, there were a total of 182 factor pairs in 2015 with a mean interacting force of about 0.69, a minimum of 0.11, and a maximum of 0.95; there were a total of 210 factor pairs in 2019 with a mean value of about 0.64, a minimum of 0.07, and a maximum of 0.9. Based on the mean interacting force of factors in 2015 and 2019, as well as the maximum and minimum values, and to promote the relative balance of the number of each type of factor pairs, the factor pairs were classified into three types of high, medium, and low level, with 0.82 and 0.88 as the threshold for $Y_{1\;}$, while the values were 0.53 and 0.57 for $\;Y_{2\;}$ in 2019, and in 2015, they were 0.84 and 0.9 for $Y_{1\;}$ while 0.6 and 0.7 for $\;Y_{2\;}$ (see Table 4, Figure 8 and Figure 9). Among them, per capita GDP ($X_{2}$), utility land ($X_{13}$), industrial smoke (dust) emission ($X_{15}$), and other factors are in strong interaction with other factors. All of the factor pairs were bifactor-enhanced or nonlinearly enhanced with each other from 2015 to 2019, with no independent or asymptotic relationships; this indicates that the factors are closely related to each other, and the combined effect of two factors on urban green space change is stronger than that of a single factor. The factor pairs were predominantly bifactor-enhanced and supplemented by nonlinear enhancement. There were 21 factor pairs nonlinearly enhanced in 2019, accounting for about 10%. There were 18 factor pairs nonlinearly enhanced in 2015, accounting for about 9.9%.

Significantly, in the interaction factor pairs of urban green area ($Y_{1}$), the interaction between urban area ($X_{5}$) and per capita GDP ($X_{2}$), and that between the urban population ($X_{4}$) and per capita GDP ($X_{2}$) were the highest in 2015, reaching 0.95 and 0.94, respectively. It indicates that at the initial stage of the study, the interaction between urban area, the urban population, and per capita GDP has the greatest promoting effect on the increase in urban green area ($Y_{1}$). In 2019, the interaction between investment completed in the current year ($X_{3}$) and per capita GDP ($X_{2}$), and that between land for commercial service facilities ($X_{10}$) and per capita GDP ($X_{2}$) were the highest: both 0.9. It indicates that as China’s urbanization development enters the stock era, the interaction between investment completed in the current year, land for commercial service facilities, and per capita GDP becomes the primary driving force for the increase in urban green area ($Y_{1}$).

In the interaction factor pairs of park area ($Y_{2}$), per capita GDP ($X_{2}$) and output value of tertiary industry ($X_{9}$) had the largest interaction of 0.84 in 2015, indicating that at the initial stage of the study, economic development is the primary driving force of the increase in park area ($Y_{2}$). In 2019, the average wage of on-job employees ($X_{6}$) and utility land ($X_{13}$) had the largest interaction of 0.71, indicating that with the improvement of people’s living standards, the interaction of average wage of on-job employees ($X_{6}$) and utility land ($X_{13}$) has the greatest promoting effect on the increase in park area ($Y_{2}$).

## 4. Discussion

### 4.1. Drive Mechanism

At the significance level of 5%, urban green space ($Y_{1}$) corresponds to the factor power (q-value) to measure the influence on urban development and supply; urban park area ($Y_{2}$) corresponds to the factor power (q-value) to measure the influence on quality of life and demand. In terms of supply, the mean influence of the 15 factors was 0.60 in 2019 and the mean influence of the 14 factors was 0.61 in 2015. In terms of demand, the mean influence of the 15 factors was 0.36 in 2019, and the mean influence of the 14 factors was 0.42 in 2015.

**Figure 10:**
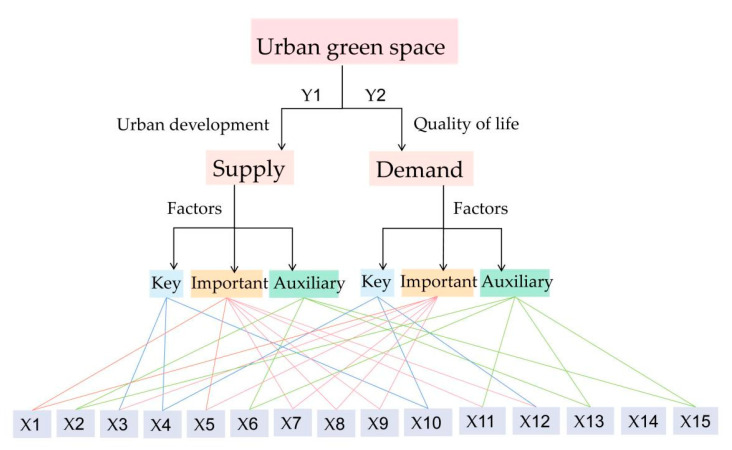
Analysis diagram of driving mechanism of urban green space change.

Based on the ranking and the mean values of the factor forces, the driving factors can be classified into three levels: that is, “Key factors”, “Important factors”, and “Auxiliary factors”. “Key factors” are dominated by the direct force, with the interaction between factors. It is judged on two grounds: first, it must be in the top three in 2019, and second, it must be above the mean value in 2015. “Important factors” work simultaneously in the form of direct force and factor interaction force. It is judged by the fact that the q-value of the factor is greater than the mean value in both 2015 and 2019. “Auxiliary factors” have a very weak direct force and are dominated by interactions, and most of them are factors with significant interactions (see Figure 10 and Table 5). The driving mechanism of spatial distribution of urban green space supply and demand is quite different, but urban population and commercial service facilities land are their key influence factors, having a comprehensive influence on the spatial–temporal changes of urban green space.

Some of the conclusions of this paper are in agreement with partial views in the existing papers, while they are in conflict with some others. These new findings are of great value for how to deal with the new challenges of urban green space construction in the new period. Similarly, Kuang [65] and Cheng [49] found that uneven development appears in urban greening among the zones, too. The existing studies have shown that socio-economic determinants [66] play an important role in the spatio-temporal change of green space. For example, GDP [60], the output value of secondary and tertiary industries [59], the urban population and urban area [64], and the average wage of on-job employees [61] are positively correlated with urban green space. Residents’ demand for ecological services provided by green space will increase with the improvement of life quality, and government investment will also directly affect the area of urban green space [66]. The difference is that among the 15 drivers involved in this paper, the urban population factor ($X_{4}$) reflecting the potential scale of urban green space demand is the strongest factor influencing changes in urban green space and park area, while in previous studies, per capita GDP is often the most important driver in the development of urban green space [58,64]. This paper argues that as urban scale and economic development flatten out and urban development gradually changes from quantitative to qualitative changes, the attractiveness of urban development quality to the urban population and the urban population’s demand for urban green space have become the most important drivers of current urban green space development. However, the interaction between per capita GDP and other factors is significant, and it can promote the change of urban green space together with other factors, just like utility land and industrial smoke. Therefore, the joint force of multiple factors should be stimulated to drive the improvement of green space construction more efficiently.

An in-depth analysis of the driving effects of the driving factors on urban green area and park area reveals that on the one hand, economic development, social conditions, industrial structure, infrastructure, and ecological environment have positive effects on both, and most of the influencing factors are at the same driving level for both urban green space and park area. On the other hand, a small number of influencing factors have some differences in influence on the two. The investment completed in the current year ($X_{3}$), industrial land ($X_{11}$), and land for road traffic facilities ($X_{12}$) are at different driving levels for urban green area and park area. The investment completed in the current year indicates that the overall input is more important for urban green area, while the land for road traffic facilities reflects that the accessibility of the park is more important for the change of park area, and the influence of industrial land on urban green area is significantly greater than that of park green area.

### 4.2. Planning and Policy Recommendations

In the past five years, cities along the Yangtze Economic Belt have carried out a series of key greening projects, including “Building a green and ecological corridor along the Yangtze Economic Belt”, “Building Garden Towns”, and “Building beautiful villages”, which have greatly improved their green areas. China is currently trying its best to establish a regional ecological and environmental control system, and the 11 provinces (cities) in the YREB region are important pilot areas. In addition, the Guidance on Strengthening Reforestation and Greening in the Yangtze River Economic Belt puts forward nine specific measures to accelerate the pace of reforestation and strengthen forest management and protection, and the National Forest City Development Plan (2018–2025) puts forward the requirement of “expanding urban green space” [67]. In general, the planning and policies for urban green space are at a broad stage.

In the pursuit of sustainable development, a balance between economic growth, environmental concerns, and social equity is essential [68,69]. The efficiency of green space utilization can be improved in general by improving land use efficiency, coordinating the economic growth of construction land with environmental protection, and adopting practical ways to transform the stock of ecological land across regions [70], to accelerate the construction of the green ecological corridor in the Yangtze River Economic Belt and the construction of the first demonstration belt of ecological civilization.

**Figure 11:**
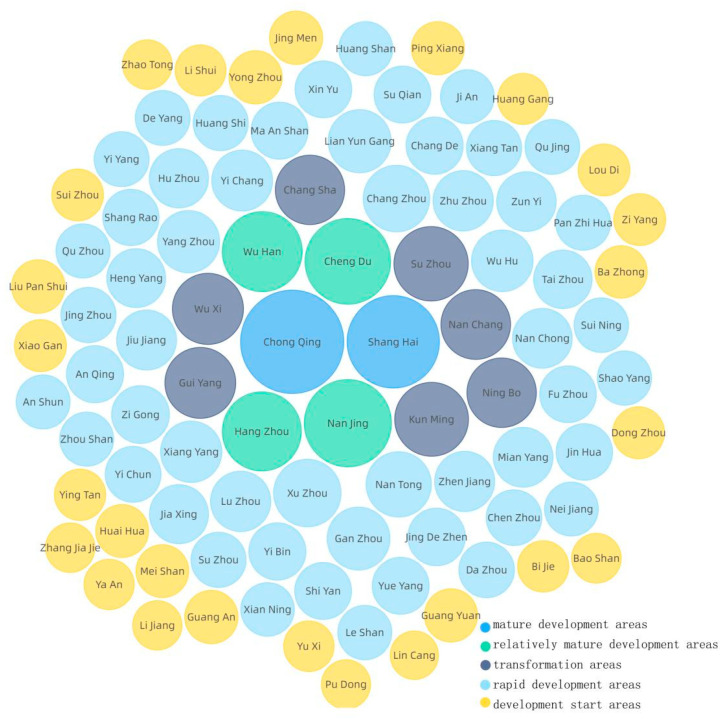
Classification of urban green space construction status.

**Figure 12:**
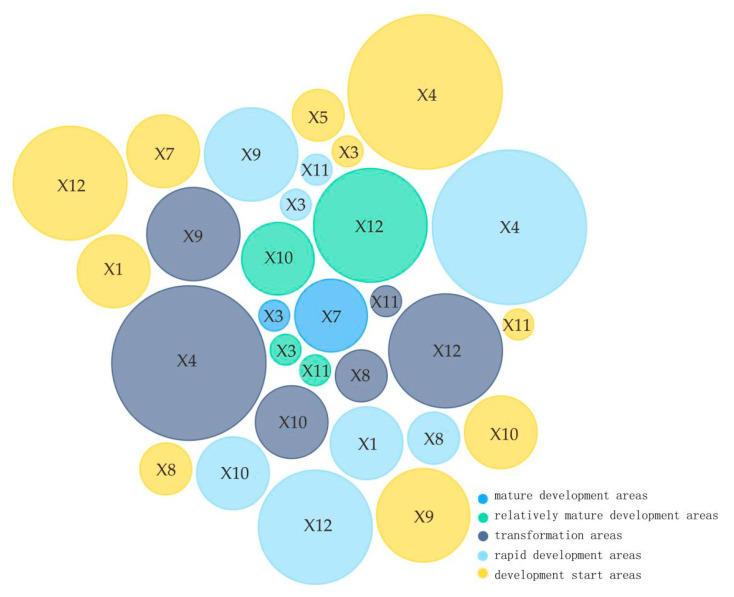
Urban green space planning and policy analysis.

In the construction of urban green space, we should make rational use of the driving effects of economic development, social conditions, industrial structure, infrastructure, and ecological environment, as well as their mutual promotions. By averaging the influence of the influencing factors on urban green spaces in 2015 and 2019, we found the forces of the influence on urban green space were ranked from largest to smallest as $X_{4}$ > $X_{12}$ > $X_{9}$ > $X_{1}$ > $X_{7}$ > $X_{10}$ > $X_{5}$ > $X_{8}$ > $X_{3}$ > $X_{11}$ > $X_{13}$ > $X_{15}$ > $X_{6}$ > $X_{2}$ > $X_{14}$. The values from $X_{4}$ to $X_{11}$ did not differ much, so they were selected as the core influencing factors. According to the patterns of spatial and temporal distribution of urban green space ranked by the average of green space area in the past five years, the current urban green space construction is classified into five types (see Figure 11): Type A consists of mature development areas, Type B consists of relatively mature development areas, Type C consists of transformation areas, Type D consists of rapid development areas, and Type E consists of development start areas, and corresponding planning and policy recommendations are proposed (see Figure 12). Type A covers Chongqing and Shanghai, two cities under the direct jurisdiction of the Central Government, where the green space is already mature, and since there is little room for expansion of the urban area, the development of their green area focuses on quality improvement, increasing government investment ($X_{3}$) and local general public budget expenditure ($X_{7}$). Type B covers four mega-cities of Nanjing, Chengdu, Wuhan, and Hangzhou, where the green space is relatively mature, and the focus should be placed on increasing government investment ($X_{3}$), optimizing the urban land structure ($X_{10},\;X_{11},X_{12}$), accelerating the construction of park cities represented by Chengdu, especially achieving the qualitative leap of park green space while quantitatively increasing the urban green space. Type C covers other provincial capitals and some eastern coastal cities, such as Suzhou, Kunming, Wuxi, Guiyang, Nanchang, Ningbo, and Changsha, where the green space is facing transformation, and efforts should be made to optimize urban land-use structure ($X_{10},X_{11},X_{12}$) and industrial structure ($X_{8}$, $X_{9}$), with a focus on the attractiveness to urban population ($X_{4}$). Type D covers central and eastern prefecture-level cities, where the green space is under rapid development, and they should increase government investment ($X_{3}$), optimize urban land use structure ($X_{10},\;X_{11}$, $X_{12}$) and industrial structure ($X_{8}$, $X_{9}$), with a focus on urban GDP growth (X1) and the attractiveness to the urban population ($X_{4}$). Type E mainly covers western prefecture-level cities, where the green space is in its infancy and has more room for development. They should accelerate the development of related urban sites ($X_{5}$, $X_{10}$, $X_{11}$, $X_{12}$), optimize the industrial structure ($X_{8}$, $X_{9}$), and increase government investment ($X_{3}$) and local general public budget ($X_{7}$) with the growth of urban GDP ($X_{1}$) and the attractiveness to urban population ($X_{4}$) as primary targets.

## 5. Conclusions

Urban green space improves the health, well-being, and aesthetics of urban residents, while playing an important role in relieving environmental pressure. However, due to the increasing demand for urban facilities as a result of rapid urbanization and continuous population growth, the development of urban green space is also facing tremendous pressure and challenges. Therefore, there is an urgent need to protect and improve existing urban green space, while developing new urban green infrastructure, which is essential for planning, management, and public health [71].

Based on the geographical spatial differences of urban green space and integrating the factors of economy, society, industry, land use, and environment, in this paper, we constructed a research framework of “space-supply-demand” integration of urban green space by the GI and geodetector methods, and we conducted an empirical study on the spatial–temporal changes of urban green space and its driving mechanism in prefecture-level cities along the Yangtze River Economic Belt in China.

The study has shown that first, the urban green space along the Yangtze River Economic Belt is concentrated in spatial distribution, while uneven development appears in urban greening among the zones. Second, the influence of different factors on urban green space change varies greatly and can be divided into three types: key factors, important factors, and auxiliary factors. The driving mechanism of spatial distribution of urban green space supply and demand is quite different, but urban population and commercial service facilities land are their key influence factors, having a comprehensive influence on the spatial–temporal changes of urban green space. Thirdly, the factors are classified into three categories of high, medium, and low levels according to the mean of interacting forces, in particular the factors of per capita GDP, utility land, industrial smoke (dust) emissions, and other factors have a very strong interactive effect with other factors. Fourth, according to the spatial distribution characteristics of urban green space and its driving mechanism, this paper puts forward policy design suggestions, providing reference for other regions in China and regions abroad to deal with green space change.

This study provides a new research framework and method for the researchers of urban and rural planning, human geography, and landscape architecture to analyze the spatial–temporal change characteristics of urban green space and its influencing factors, and it helps reveal the development law and driving mechanism of urban green space, with great theoretical value. It also provides a valuable reference for government decision making and urban planning, and it is of great practical significance for the protection of urban green space ecosystem along the Yangtze River Economic Belt in China. The research methods and conclusions of this paper also provide an important reference for the study of urban green space change at urban and regional scales. The shortcoming is that in addition to the 15 driving factors in this study, the implementation of government policies and regulations on urban planning, real estate development, and environmental protection is another major factor affecting the spatial–temporal change of urban green space [72], while in this study, we took policies as a potential factor affecting social economy and green space. It limits the power of the findings to explain the spatial–temporal change of urban green space in prefecture-level cities along the Yangtze River Economic Belt in China, and further research in this area is needed in the future.

## Figures and Tables

**Table 1:** Types of Gini index.

Gini Index	Meaning	Numerical Representation in this Paper
0.0–0.2	Absolutely even	Quite evenly distributed
0.2–0.4	Even	Evenly distributed
0.4–0.6	A large gap	Concentrated distribution
0.6–1.0	A great gap	Rather concentrated distribution

**Table 2:** Types of interaction: interaction between explanatory variables ($X_{i}$ and $X_{j}$).

Description	Interaction
$q(X_{i}\cap X_{j})\;<\;\mathrm{Min}(q(X_{i}),\;q(X_{j}))$	Weaken, nonlinear
$\mathrm{Min}(q(X_{i}),\;q(X_{j}))\;<\;q(X_{i}\cap X_{j})\;<\;\mathrm{Max}(q(X_{i})),\;q(X_{j}))$	Weaken, univariate
$q(X_{i}\cap X_{j})\;>\;\mathrm{Max}(q(X_{i}),\;q(X_{j}))$	Enhance, bivariate
$q(X_{i}\cap X_{j})\;=\;q(X_{i})+q(X_{j})$	Independent
$q(X_{i}\cap X_{j})\;>\;q(X_{i})\;+\;q(X_{j})$	Enhance, nonlinear

**Table 3:** Model variable description.

Variable	Index	Code	Type
Dependent variable $(Y_{i}$)	Urban green area	$Y_{1}$	Supply (urban development)
	Park area	$Y_{2}$	Demand (quality of life)
Independent variable ($X_{i}$)	Gross domestic product (GDP)	$X_{1}$	Economic driving force
	Per capita GDP (GDPPC)	$X_{2}$	
	Investment completed in the current year	$X_{3}$	
	The urban population	$X_{4}$	Social driving force
	Urban area	$X_{5}$	
	Average wage of on-job employees	$X_{6}$	
	Local general public budget expenditure	$X_{7}$	
	Output value of secondary industry	$X_{8}$	Industrial structure driving force
	Output value of tertiary industry	$X_{9}$	
	Land for commercial service facilities	$X_{10}$	Land-use structure driving force
	Industrial land	$X_{11}$	
	Land for road transportation facilities	$X_{12}$	
	Utility land	$X_{13}$	
	Density of drainage pipe in built-up area	$X_{14}$	Ecological environment driving force
	Industrial smoke (dust) emission	$X_{15}$	

**Table 4:** Statistical analysis of factor pairs and interaction forces.

		Number of Factor Pairs				Strength of Interaction Effect			Legend		
		Total	High	Medium	Low	Min	Max	Average	High	Medium	Low
2019	$Y_{1}$	105	11	40	54	0.07	0.90	0.79	>0.88	0.82–0.88	<0.82
	$Y_{2}$	105	14	23	68	0.07	0.71	0.50	>0.57	0.53–0.57	<0.53
2015	$Y_{1}$	91	13	24	54	0.27	0.95	0.80	>0.90	0.84–0.90	<0.84
	$Y_{2}$	91	11	30	50	0.11	0.84	0.58	>0.71	0.60–0.71	<0.60

**Table 5:** Classify the driving factors: blue represents “Key factors”, orange represents “Important factors”, and green represents “Auxiliary factors”.

Number	Supply: Urban Development (*Y*_1_)				Demand: Quality of Life (*Y*_2_)			
	2019		2015		2019		2015	
1	$X_{10}$	0.77	$X_{4}$	0.76	$X_{10}$	0.49	$X_{4}$	0.53
2	$X_{4}$	0.77	$X_{1}$	0.74	$X_{4}$	0.48	$X_{5}$	0.52
3	$X_{3}$	0.76	$X_{9}$	0.74	$X_{12}$	0.47	$X_{12}$	0.51
4	$X_{12}$	0.76	$X_{12}$	0.73	$X_{5}$	0.46	$X_{9}$	0.49
5	$X_{9}$	0.76	$X_{8}$	0.73	$X_{3}$	0.45	$X_{7}$	0.48
6	$X_{1}$	0.75	$X_{7}$	0.72	$X_{7}$	0.45	$X_{1}$	0.48
7	$X_{5}$	0.75	$X_{11}$	0.71	$X_{9}$	0.44	$X_{11}$	0.46
8	$X_{7}$	0.74	$X_{10}$	0.68	$X_{1}$	0.43	$X_{8}$	0.46
9	$X_{11}$	0.74	$X_{3}$	0.66	$X_{8}$	0.42	$X_{10}$	0.45
10	$X_{8}$	0.73	$X_{5}$	0.63	$X_{11}$	0.41	$X_{3}$	0.45
11	$X_{13}$	0.51	$X_{13}$	0.43	$X_{13}$	0.29	$X_{15}$	0.33
12	$X_{2}$	0.33	$X_{15}$	0.38	$X_{6}$	0.19	$X_{13}$	0.31
13	$X_{6}$	0.28	$X_{6}$	0.35	$X_{15}$	0.18	$X_{6}$	0.24
14	$X_{15}$	0.22	$X_{2}$	0.27	$X_{2}$	0.08	$X_{2}$	0.11
15	$X_{14}$	0.07			$X_{14}$	0.07		

## Data Availability

The data used in this paper mainly come from the China City Statistics.
